# Empowering Foot Health: Harnessing the Adaptive Weighted Sub-Gradient Convolutional Neural Network for Diabetic Foot Ulcer Classification

**DOI:** 10.3390/diagnostics13172831

**Published:** 2023-09-01

**Authors:** Abdullah Alqahtani, Shtwai Alsubai, Mohamudha Parveen Rahamathulla, Abdu Gumaei, Mohemmed Sha, Yu-Dong Zhang, Muhammad Attique Khan

**Affiliations:** 1 Department of Software Engineering, College of Computer Engineering and Sciences, Prince Sattam bin Abdulaziz University, Al-Kharj 11942, Saudi Arabia; ms.mohamed@psau.edu.sa; 2 Department of Computer Science, College of Computer Engineering and Sciences, Prince Sattam bin Abdulaziz University, Al-Kharj 11942, Saudi Arabia; sa.alsubai@psau.edu.sa (S.A.); a.gumaei@psau.edu.sa (A.G.); 3 School of Podiatric Medicine, The University of Texas Rio Grande Valley, Harlingen, TX 78550, USA; mohamudha.parveen@utrgv.edu; 4 Department of Basic Medical Sciences, College of Medicine, Prince Sattam bin Abdulaziz University, Al-Kharj 11942, Saudi Arabia; 5 School of Computing and Mathematical Sciences, University of Leicester, Leicester LE1 7RH, UK; 6 Department of CS, HITEC University, Taxila 47080, Pakistan; attique.khan@hitecuni.edu.pk; 7 Department of Computer Science and Mathematics, Lebanese American University, Beirut 1102-2801, Lebanon

**Keywords:** diabetic foot ulcer, deep learning, random initialization of weights, convolutional neural network, Adaptive Sub-gradient Optimizer

## Abstract

In recent times, DFU (diabetic foot ulcer) has become a universal health problem that affects many diabetes patients severely. DFU requires immediate proper treatment to avert amputation. Clinical examination of DFU is a tedious process and complex in nature. Concurrently, DL (deep learning) methodologies can show prominent outcomes in the classification of DFU because of their efficient learning capacity. Though traditional systems have tried using DL-based models to procure better performance, there is room for enhancement in accuracy. Therefore, the present study uses the AWSg-CNN (Adaptive Weighted Sub-gradient Convolutional Neural Network) method to classify DFU. A DFUC dataset is considered, and several processes are involved in the present study. Initially, the proposed method starts with pre-processing, excluding inconsistent and missing data, to enhance dataset quality and accuracy. Further, for classification, the proposed method utilizes the process of RIW (random initialization of weights) and log softmax with the ASGO (Adaptive Sub-gradient Optimizer) for effective performance. In this process, RIW efficiently learns the shift of feature space between the convolutional layers. To evade the underflow of gradients, the log softmax function is used. When logging softmax with the ASGO is used for the activation function, the gradient steps are controlled. An adaptive modification of the proximal function simplifies the learning rate significantly, and optimal proximal functions are produced. Due to such merits, the proposed method can perform better classification. The predicted results are displayed on the webpage through the HTML, CSS, and Flask frameworks. The effectiveness of the proposed system is evaluated with accuracy, recall, F1-score, and precision to confirm its effectual performance.

## 1. Introduction

Numerous diabetes-infected individuals are likely to develop DFUs that cause maximum amputations [1], typically due to deprived glycemic control, peripheral vascular disease, underlying neuropathy, or meager foot care. Moreover, DFU can happen at all ages; however, it seems to be common among people aged around 45 years. Nevertheless, the conventional diagnosis process for DFU by medical experts and specialists is time-consuming and expensive [2]. Hence, DL-based approaches in healthcare imaging clear the path for automatic analysis of DFU. Provided with the complex form of DFUs, the AI techniques seem appropriate for addressing certain significant aspects like time screening for identifying foot ulcer risk in accordance with suitable sensor technologies [3,4]. Considering this, existing studies have attempted to use different DL-based models for DFU prediction. Accordingly, one study [5] used a stacked parallel convolutional layer with appropriate intermediate integrations of transition, convolution, and max-pooling layers relying on the CNN to determine DFU in classifying abnormal and normal DFU skin patches. In the DFU images, the normal images appear to be small and are usually red or pink in color without any abnormality. Whereas the abnormal images look like inflammation, which can be indicated as a red area in the image, which is present with abnormality. In biomedical imaging, abnormal images depend upon the intensity of the pixels in an image that are used to identify abnormalities. For example, a tumor may appear brighter or darker than the surrounding tissue.

Various filter sizes in the intermediate layers and parallel convolutional layers among individual parallel blocks have assisted in retrieving significant features from input images. In conv2d_1 = (none, 75, 75, 32), conv2d_2 = (none, 38, 38, 64), conv2d_3 = (none, 19, 19, 128) layers. In max_pooling2d = (none, 38, 38, 32), max_pooling2d_1 = (none, 19, 19, 64), max_pooling2d_3 = (none, 10, 10, 128). The convolutional neural networks (CNNs) consist of 2D matrix features where each of the features is weighted as a sum of the input pixels. In this CNN, there are several attributes that can possibly affect the precision of the CNN, including the size and complexity of the model, the quality of the training data, and the optimization algorithm used during training. In a deep neural network with some level of complexity, there are usually at least two layers involved in qualifying it as a deep neural network (DNN), or deep net for short. Deep nets are used in processing the data via complex ways by employing sophisticated mathematical modeling. Furthermore, natural data augmentation for enhancing the training images supports resolving restricted training data-oriented issues. The suggested DFU_SPNet was trained using multiple learning rate settings, and optimizers showed better performance with 0.974 as the AUC rate.

Further, one paper [6] utilized a DL methodology for localizing and classifying DFU. Sixteen layers of the suggested CNN model achieved better classification outcomes relying on the integration of the chosen convolutional layers. The Yolo-v2 DFU model was designed to perform localization using ShuffleNet as the backbone for Yolo-v2. Localization outcomes confirmed that integration of Yolo-v2 and the ShuffleNet model was satisfactory in determining the infected area of foot images. Further, for better use of the knowledge within training data, CKBs (Class Knowledge Banks) have been endorsed. This has encompassed trainable units, which can better extract and indicate class knowledge. An individual CKB unit has been utilized for similarity computation with representation retrieved from the input image. The average similarity among units within a CKB and their indication can be considered a logit of regarded input. In this manner, prognostication relies upon the input images, trained network parameters, and the CK attained from training data and maintained in CKBs. Empirical outcomes have revealed that the suggested methodology can satisfactorily enhance the classification of ischemia and DFU infection [7].

Further, research [8] has suggested a smartphone-oriented skin telemonitoring system for assisting in medical decisions and diagnosis while analyzing DFU tissues. The database encompassed 219 images. Moreover, for optimal identification of tissue and ground truth annotation, a graphical interface relying on the super-pixel segmentation methodology was utilized. The suggested method performed DFU analysis in an end-to-end format encompassing automatic segmentation of ulcers and classification of tissue. Classification was performed in a patch-wise manner. The super-pixels retrieved with SLIC were utilized as an input to train a DNN (deep neural network). Conventional DL-based models to perform semantic-wise segmentation were utilized to differentiate tissues within ulcer regions into three main classes (slough, granulation, and necrosis). A comparison was performed with the suggested methodology. The suggested super-pixel-oriented method outperformed traditional full CNN models. The accuracy value improved from 84% to 92%, whereas the DICE index was enhanced from 54% to 75%. Outcomes exposed robust classification and the potential for monitoring DFU healing.

To enhance the accuracy rate, one study [9] recommended using ResNet-101 and AlexNet to classify foot images for detecting DFU. The foot image dataset encompassing healthy skin and DFUs was attained from the Kaggle dataset. Various epochs and dataset split-ratios were applied to assess the ideal performance of the suggested model. Better outcomes were attained at 40 epochs when the (80:20) ratio was considered. AlexNet achieved 97.1% accuracy, whereas ResNet achieved 97.1% accuracy. Such frameworks have exposed optimal outcomes and will assist in better diagnosis of DFU.

Furthermore, FusionSegNet has been suggested, integrating local wound features and global foot features, for identifying DF-based images from the DFU image dataset. FusionSegNet integrated two types of features for making final predictions. During training and validation, 1211 images were gathered for fivefold cross-validation. The endorsed system classified the non-DF and DF images with a better AUC (area under receiver operating characteristics) rate. With such an ideal performance, the suggested methodology can extract the wound features better, thereby improving classification performance [10]. Though conventional models have endeavored to work better in DFU classification, there is a scope for enhancement in accordance with the accuracy rate. The advantage of the proposed approach, which is based on image analysis, compared to an approach using biochemical parameters is that it can be more quantitative than the image analysis method regarding the biochemical parameters. The advantage of two approaches is that image analysis is used to detect subtle changes that may not be visible with biochemical parameters. It can be used to analyze samples in real time when compared to an approach using biochemical parameters. The disadvantage of two approaches is that image analysis can be more time-consuming and labor-intensive than biochemical methods. It requires more specialized equipment and software than biochemical parameters. Image analysis may not be as sensitive as biochemical methods for detecting certain changes. The reasons for the proposal with respect to the state of the art are deliberated, and how the proposal surpasses the other approaches is deliberated and highlighted for reference.

Both random initialization weight (RIW) and Adaptive Sub-gradient Optimizer (ASGO) algorithms are used in the proposed model. The main reasons for implementing RIW and ASGO are as follows. RIW is used to initialize the weights of a neural network where the weights can be initialized to random values, which helps to prevent the network from getting stuck in local minima during training. It can also improve the generalization performance of the network. RIW is especially used as high-dimensional data for images, which denotes that there are a large number of possible weights. It also helps to ensure that the network explores a wide range of possible weights, achieves a good result, and learns the shift of feature space between the convolutional layers. Further, the ASGO is used in tracking the sum of the squares of the gradients for each feature. It is a variant of the gradient descent algorithm that adapts the learning rate for each parameter individually. The ASGO uses the log softmax function, and when logging softmax with the ASGO is deployed for the activation function, the gradient steps are controlled. An adaptive modification of the proximal function simplifies the learning rate significantly, and optimal proximal functions are produced. Thus, the proposed model possesses the ability to generalize to new data by handling noise or outliers and can enable interpretability or explainability. The other approaches used in DFU classification can be sensitive to noise and outliers, which can lead to inaccurate results and tends to increase complexity and time-consumption in extracting features and classification of normal and abnormal DFU. Whereas the proposed model is not disposed to noise and other outliers, which can lead to more accurate results. In the random initialization of the weights, it helps to prevent the algorithm from overfitting the training data. Hence, the proposed method is found to be efficient and easier to tune and produces less computational complexity.

Considering accuracy and the F1-score as the significant parameters, the present study intends to attain a high classification rate using the proposed algorithms based on the below objectives.

The significant contributions of this study are:

- To classify the normal and abnormal DFU images using the proposed AWSg-CNN (Adaptive Weighted Sub-gradient Convolutional Neural Network) for attaining a high prediction rate;
- To deploy a Flask model for easy creation of web applications to predict foot ulcers, thereby displaying remedies in abnormal cases;
- To evaluate the proposed model by comparison with conventional methods for exposing the ideal performance of the model in DFU classification.

### Paper Organization

The paper is organized as follows. Section 2 reviews traditional models with relevant problem identification. This is followed by Section 3, with an overview of the proposed system with proper flow, algorithm explanation, and processes. The outcomes attained from the execution of the proposed model are discussed in Section 4. Lastly, the study is concluded in Section 5 with future directions.

## 2. Review of Existing Work

Existing research works have strived to perform better DFU classification using ML- and DL-based models. Problems corresponding to these works are discussed in this section.

ML models have gained attention in DFU classification [11]. In accordance with this, one study [12] considered ML for DFUs in the inpatient population. Global inpatient samples were assessed from 2008 to 2014. Chi-square testing was undertaken with the use of variables that were positively associated with DFUs. To perform descriptive measurements, the Wilcoxon rank-sum test, chi-square test, and Student’s *t* test were utilized. Six predictive features were determined. A DT (decision tree) framework, namely, CTREE, was used to assist in developing the algorithm. The model’s performance in six variable tests was 79.5%. The AUC value for the 6-variable framework was 0.88. The suggested model showed an accuracy rate of 79.8%.

Further, the research [13] compared an ML-based scoring methodology with optimization and feature selection methods. Comparatively trivial CNN models, namely, MobileNetV2, showed 95% as an F1-score for 2-foot thermogram image-oriented classification, while AdaBoost utilized 10 features and thereby achieved 97% as an F1-score. Comparing the inference times for ideally performing networks confirmed that the suggested method could be provided as a smartphone application, permitting the user to monitor the DFU progression within the home setting.

Furthermore, one study [14] utilized Danish National Registration data to assess the risk factors in diabetes patients for developing DFU and experiencing amputation. It assessed 246,705 patient data. The medical records of individuals and socioeconomic information were researched. Factors like cardiovascular disorders, chronic renal complexities, low disposable income, peripheral artery disease, and neuropathy were among the significant risk factors. Depression and mental disorders still comprised the maximum risks compared to individuals without such complexities.

Furthermore, ML methods have been considered for assessing the practical usage of these risk factors to prognosticate amputation and foot ulcers. Risk analysis of socioeconomic and medical features represented a maximum hazard of experiencing amputation or DFU for patients with cardiovascular disease, neuropathy, chronic renal complexities, and peripheral vein disease. Recently, DFU classification was considered with DL in addition to ML [15]. In the area of DFU analysis, DL-based models are being used by investigators for recognizing and detecting DFU.

In accordance with this, one study [16] suggested using the DFINET (Diabetic Foot Infection Network) for assessing non-infection and infection from DFU images. It has a similar ReLU convolution with 22 layers, a normalization layer, and a dropout connection of interconnected layers. The results showed that when the DFINET is integrated with the enhanced augmentation image, the accuracy rate is 91.98%, and the binary arrangement of the Matthews correlation coefficient is improved to 0.84. The enhancement of these techniques revealed that the methods might support medical professionals in detecting DFU automatically. Moreover, the authors of [17] emphasized automatic detection of DFU in the dataset of the Diabetic Foot Ulcer Challenge (DFUC), which has 4500 images of DFUs. Aimed at identifying the problem, the study used an architecture for object detection called the EfficientDet method to eliminate overlapping bounding boxes and score thresholds. Additionally, modification of the network predictions arranged the test dataset by threshold score and eliminated the overlapping bounding boxes. As a result of the EfficientDet method, there exists an elimination of the overlapping bounding boxes and score threshold.

In addition, another study [18] dealt with the synthesis of ML with custom-made lower-level CNNs and higher-level structures to improve DFU automated diagnosis. Similarly, an original CNN architecture was projected as stated in residual blocks on removing higher-level structures. Synthesis structures of various ML models such as support vector networks, gradient boosting, and neural networks have improved identifying DFU results associated with the simulated characteristic grouping. Thus, LRC demonstrated that the outcomes of entire estimation standards can attain a 95.6% sensitivity and an AUC value of 96.50%. Furthermore, one study [19] designed and executed a robust deep CNN model, namely, DFU_QUTNet, in accordance with the idea of enhancing the width of the network with global average pooling. A dataset was gathered of 754 patients with healthy skin and DFU. The features retrieved from the suggested model were utilized for training KNN and SVM classifiers. The performance of the endorsed network was compared with conventional CNN networks, namely, AlexNet, VGG-16, and GoogleNet. The performance for classifying DFU enhanced the F1-score at a rate of 94.5%. To determine the existence of ischemia and infection in DFU, a feature descriptor termed the super-pixel color descriptor was used [20].

Subsequently, ensemble CNN was utilized to better recognize infection and ischemia. A natural data augmentation technique was suggested to identify the RoI (region of interest) on the foot images and concentrated on determining the salient features in this sector. It was determined that the suggested ensemble CNN model performed better, with 73% accuracy in classifying infection and 90% accuracy in classifying ischemia. Additional research [21] also compared four hybrid CNN models for automated DFU classification. Initially, the suggested models were trained with the original images. Following this, the training was provided with actual images and augmented images. Outcomes revealed that a model with four branches showed better outcomes than two, three, or five branches in DFU classification by achieving 95.8% as the F1-score rate. The authors of [22] employed DFU_VIRNet with dual kinds of diabetes foot samples, infrared images, and visible images. The accuracy value is 97.7%.

Further, the authors of [23] considered an ensemble model for categorizing wound images (including venous, surgical, and diabetic ulcers). The resultant classification rate of dual classifiers was fed into an MLP (Multi-Layer Perceptron). Classification accuracy was 94.28% and 96.4% for binary. Furthermore, the authors of [24] aimed to classify the sequences in patients’ thermal images. Common structures were tested on the transfer learning mode, including GoogleNet and AlexNet. In addition, a DL model was designed to reach maximum accuracy rates. Comparisons were performed with traditional classifiers such as SVMs, ANNs, and CNNs. Outcomes of initial simulations with conventional SVM and ANN classifiers attained satisfactory outcomes after feature extraction. To enhance system performance, a CNN model was preferred to discriminate among five diabetes severity and non-diabetes severity grades from the thermal images. The recommended model showed ideal performance with a 0.98 accuracy rate. The outcomes revealed that e-data augmentation is appropriate for detecting and grading diabetic subjects.

On the contrary, another study [25] attempted to evaluate the procedures for determining if the patient possesses a foot ulcer. Varied DL-based models were executed for predicting ulcers, namely, VGG-16, MobileNet, and ResNet. After the analysis, VGG-16 was better than the equivalent methods in diagnosing DFU [26]. Further, as an enhancement, a faster RCNN was used with suitable parameter settings. Training was assessed with the Monte Carlo cross-validation methodology. The suggested system achieved a mean precision rate of 91.4% and an F1-score rate of 94.8% [27]. Moreover, one study [28] attempted to develop a smart prognosis model for DFU in accordance with DL for conducting preliminary analysis. RetinaNet Scale and Mask RCNN were utilized for model construction. Better outcomes were attained.

This suggested approach has the main aim of assessing the impact of glucose control, diabetes-related complications, and cardiometabolic risk factors, on the effects of diabetic foot ulcers (DFUs), especially in patients with T2D. This approach used Albanian adult inpatients with T2D for the DFU examination. The study suggested that a longer duration of diabetes, cigarette smoking, lower HDL-cholesterol levels, poor glucose control, and elevated triglyceride and SBP are some of the primary reasons for DFU. As a result, community interventions and health policies were aimed at improving the management of diabetes, which are related to resolving the cardiometabolic risk factors in Albania in order to prevent DFUs and other diabetes complications in patients with T2D [29].

The DF prediction is performed by genetic programming, which is adapted using the X-GPC. This is used in providing the global interpretation of the DFU diagnosis by using a mathematical model. A 3D graph is also procured in the suggested study, which can be used by the medical staff in evaluating the patient’s situation. This study adapted a real-world dataset in examining and diagnosing the DFU [30].

Contrarily, a cloud-based DL model and cross-platform mobile application were used for detecting DFU. The dataset was attained over six months of analysis time and utilized for retraining the conventional model to enhance its capability in detecting DFU at several phases of progress. The model was trained with a heterogeneous dataset encompassing a non-standardized form of DFU images. The clinicians chronicled agreement with nearly 178 cases, while disagreement occurred with 25 cases, leading to an overall value of 87.6% [31]. Furthermore, analyzing DFU was performed. Deep CNN-based ResKNet was suggested for performing such an evaluation. The suggested network has sequences of distinct residual frameworks of 2D-convolution, Leaky ReLU, and batch normalization. Empirically, it was proven that a shallow network with four distinct ResNets could achieve better performance [32].

### Problem Identification

Significant issues that were determined through the evaluation of conventional methods are discussed in this section.

- Existing models have attempted to perform better classification of DFU. In accordance with this, one study [12] utilized a DT model named CTREE and showed a 79.8% accuracy rate; another [13] achieved 97% as an F1-score. The authors of [16] utilized DFINET and showed a 91.9% accuracy rate; a study [21] considered four parallel branches of convolutional layers, showing a 95.8% accuracy rate; another study [22] utilized DFU_VIRNet and showed 0.977 as the accuracy rate. Furthermore, [31] considered a cloud-based DL model and cross-platform mobile application, showing an 87.6% accuracy rate. Despite the attempts of conventional models at DFU classification, there is an opportunity for further improvement.
- With a balanced dataset and enhanced data seizing of DFU, the performance of such methodologies will be enhanced in the future. Furthermore, hyper-parameter optimization of conventional ML and DL methodologies could enhance the performance rate of techniques [20].
- The classification rate and performance of the suggested methodology could be improved by attempting to use different integrations of image-wise and patch-wise classifiers [23].

Concurrently, the Deep Feature Utilization Viral Neural Network (DFU_VIRNer) refers to the model’s ability to learn long-range dependencies between the nucleotides in a viral read, and this is achieved through the use of a deep attention architecture. It has been effective in identifying a variety of viruses, including influenza and HIV. This model is capable of getting stuck in local minima and will not be able to learn the optimal parameters. But the random initialization that is used in the proposed model helps to prevent the limitations provided by DFU_VIRNer by randomly initializing the parameters of the model and improving its learning rate.

There are some benefits in our proposed approach in terms of learning time and computational complexity when compared with existing approaches.

The proposed method tends to prevent the vanishing gradient problem. When the weights are initialized to very small values, the gradients can become very small, which can make it difficult for the network to learn. Random initialization helps to prevent this problem by ensuring that the weights are not too small; the learning time of the proposed method is 8 s and the computational complexity is O(log n). Random initialization of weights can help to reduce computational complexity by making it easier for the network to converge.

In terms of learning time and computational complexity, ASGOs can typically achieve better results than traditional gradient descent algorithms. This is because they are able to converge more quickly and also update the weights that are most important for the current iteration.

## 3. Proposed Methodology

**Figure 1:**
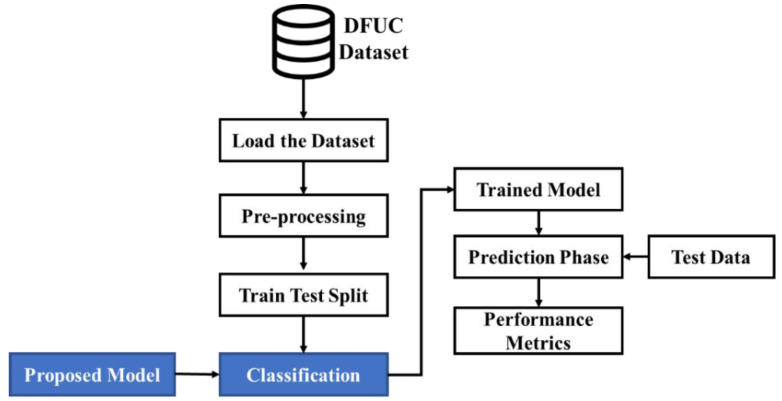
An overview of the Proposed System.

This study will classify DFU as normal or abnormal using suitable DL-based models. Though conventional systems have tried to perform better, there is room for further improvement in accordance with the prediction rate. Considering this, the present research proposes sequential processes as depicted in Figure 1. As shown in Figure 1, the dataset is loaded. Initially, the image data provided as input are pre-processed. Then the data are split into training and test data in a ratio of 80:20. Subsequently, the proposed model used for performing classification classifies the image data into normal and abnormal cases of images in the prediction phase. The viability of the classification performed is measured using performance metrics. The doctors do not need to derive each of the features from the image data. The model itself separates the features using the pre-processing phase of the image. This process provides the physicians with the needed features for examining the foot ulcer. During this process, the features will be converted into an array, and that array will be passed into the proposed model for the training and tested, and in backend, the model is classified as normal or abnormal. In our proposed system, the process of random initialization aids in preventing the model from a case of overfitting due to the presence of noise in the data. This is done when making the random weights distribute the error evenly across every feature. This can be done rather than focusing on the small number of features that are correlated with the noise. In the case of using ASG algorithms, they can be used with any type of data, as long as the data can be represented as a stream of examples. This is more flexible than approaches that rely on automatic feature extractors.

As an initial phase, data are pre-processed to eliminate inconsistent or missing data. Through this process, the quality and accuracy of the dataset can be improved, making it highly reliable. This permits the data to be consistent. The pre-processed data are fed into a training–testing split with 80% training and 20% testing. The image is amplified by applying a pre-processing technique to enhance the training images. Training has been provided with actual images and augmented images with 80% of the training dataset and 20% of the testing dataset considered. The training data are used for classification and the testing data are used for validating the performance. Subsequently, classification is undertaken with the AWSg-CNN. The proposed classic encompasses RIW (random initialization of weights) and the ASGO (Adaptive Sub-gradient Optimizer) for optimal performance. In this case, RIW is utilized to effectively learn the feature space shifts among the convolutional layers, while the logging softmax operation is utilized to avoid gradient underflow. Moreover, the ASGO is utilized for the activation function that manages the gradient phases. An adaptive modification of the proximal operation simplifies the learning rate and affords ideal proximal functions. Predictions made through the proposed classifier are evaluated regarding performance metrics to prove their efficacy.

**Figure 2:**
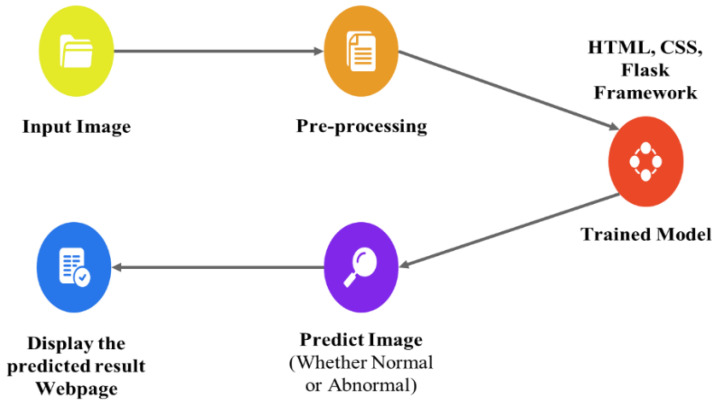
Deployment of the Flask Model for Predicting Foot Ulcer.

The proposed technique predicts outcomes that are projected on the webpage using the Flask model, HTML, and CSS with its stepwise procedures (as shown in Figure 2). In this case, the pre-processed input image is fed into the trained model to predict the image as abnormal or normal. The corresponding outcomes are displayed on the webpage. As an added merit, equivalent remedies for abnormal cases are also displayed on the webpage.

**Figure 3:**
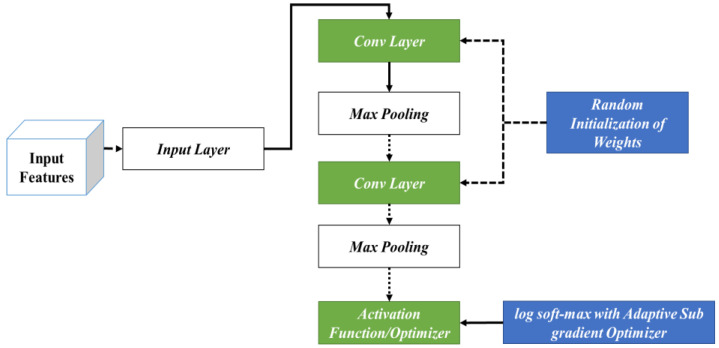
Working Procedure of the AWSg-CNN.

The specific processes considered during the classification phase are shown in Figure 3. In this phase, the input features are fed into the initial phase of the input layer. Following this, the corresponding outcomes are fed into different layers of the CNN encompassing the convolutional and max-pooling layers. In this stage, initially, RIW is performed to ideally learn the feature space shift among the convolutional layers. In addition, log softmax with the ASGO is utilized to control the gradient steps. This is then fed into the activation function for attaining optimal outcomes.

### 3.1. RIW (Random Initialization of Weights)

DL-based models feature numerous hidden neurons. Nevertheless, the main drawback of such models is the long time needed for training. Attaining an ideal accuracy rate and better training time seems challenging for the DL research community. Choosing a suitable weight initialization method is crucial while training the DL methods. Typically, weight initialization indicates the manner of setting the initial weights of the neural network. DL methodologies are sensitive to initial weight values. Weight initialization seeks to support establishing stable NN (neural network) learning bias, thereby alleviating the convergence time. Further, weight initialization is crucial in enhancing the training phase of DL-based models. The aim of the initialization of weight lies in preventing layer activation results from vanishing or exploding during DL model training.

Moreover, network training without valuable weight initialization could result in less convergence or the inability to converge. In addition, optimal weight initialization assists the gradient-based techniques with quick convergence. For comprehending the significance of the initialization of weights, it is initially vital to understand the units or neurons that make up individual CNN layers. Such neurons consider the input and perform computations to accomplish weighted summation. Then, results are generated through the activation function. Each of the neurons comprises bias and weights. Initially, weight initialization is performed, and it is assigned in accordance with the size of the input, whereas bias optimization is performed throughout the training stage.

Further, weight initializers regulate the initial weights of the network layers. The weight initialization process aims to maintain the outputs of the activation layer away from general issues of exploding and gradient vanishing. Particularly, the vanishing gradient occurs due to the backpropagation at the training stage. Feedback signal propagation from output-loss to initial layers might impact it, while signals might become lost or weak, making the network untrainable. Hence, weight setting has to be carefully undertaken to accomplish ideal outcomes and performance.

Generally, three initialization methods exist. The initial phase encompasses constant techniques that apply similar weights for initializing the network connections, like one and zero initializers. Nevertheless, while utilizing such initialization methodologies, the learning algorithm equation often becomes incapable of updating or altering the network weights, resulting in the model becoming locked. For each iteration, all the layers possess similar weights, thereby performing identical computations. The second stage includes the distribution techniques for performing the initialization, where the uniform or Gaussian distribution is employed, and input matrices are allocated with some random values. Nonetheless, assigning suitable network parameters inclusive of standard deviation and mean might indirectly impact the training performance of the model and result in vanishing gradient issues.

The last phase is random initialization in accordance with prior knowledge. For initializing the weights of the layer, heuristics are employed with non-linear activation. In this case, heuristics are employed to define the strategy to resolve the issues without utilizing the method, confirming the ideal solution. With the use of such randomization, normal distribution alteration is assigned in accordance with the inputs. Overall, RIW with prior knowledge assists as an optimal point for the initialization of weights. The main merit of such initialization involves its ability to initialize layer weights randomly, minimizing the opportunities for becoming trapped in exploding and vanishing gradient issues, avoiding low convergence speed, and alleviating the minima oscillation. Due to these advantages, the present study considers this process in the classification phase. Its overall working process is shown in Algorithm 1.
**Algorithm 1.** RIW in CNN layer.1. Initialize the weights of the convolutional layers randomly using a normal distribution with mean 0 and standard deviation 1 with a specified shape for the weight tensor.  2. Pass the input data through the convolutional layers to generate a set of feature maps.  3. Employ a non-linear activation function, such as ReLU, to the feature maps.  4. Pass the output of the initial convolutional layer to the second convolutional layer.  5. Initialize the weights of the subsequent convolutional layer using a normal distribution with mean 0 and standard deviation 1.  6. To assist in learning the shift of feature space among the first and second convolutional layers, include a bias term to the weights of the second convolutional layer proportional to the mean of the output feature maps from the first convolutional layer.  7. Iterate steps 2–6 for subsequent convolutional layers in the model.  8. Train the model with backpropagation to adjust the weights and biases of the convolutional layers in accordance with the error between the predicted output and the true output.  9. Repeat the training process with varied RIWs to avoid getting stuck in local optima.  10. Assess the model performance on a validation set to determine if it is capable of effectively learning the shift of feature space between the convolutional layers.

When the second convolution layer with the weights (W2), bias term (b2), and input (x) from the previous layer, the output of the layer is represented by Equation (1),

(1) y=activation(W2∗x+b2)

In Equation (1), activation indicates the non-linear activation operation like ReLU, and y indicates the output from the previous layer. For the execution of RIW with the bias shift, the bias term is added to the weights (W2) in proportion to the mean of the resultant feature maps from the previous layer. It can be given by Equation (2),

(2) W2=W2+alpha∗mean(x)

In Equation (2), alpha represents the hyperparameter that manages the bias shift strength, and mean(x) denotes the resultant feature maps from the previous layer.

### 3.2. Log Softmax with ASGO

The log softmax operation is a renowned activation function utilized in ML, especially during classification. It translates the vectors of the real numbers into probability distribution upon classes by employing the softmax function, followed by taking the logarithm. The ASGO is an optimization approach utilized for training NNs. Such approaches are stochastic-gradient-based methodologies that also include an altering data-reliant pre-conditioner. Their empirical accomplishment seems better due to their innate ability to converge to local optimality. Though the significance of probability distribution seems to be known, all the recent applications utilize simple probability distributions like global or uniform popularity that demand huge sizes of samples for accomplishing acceptable bias. The main cause for such inclination could be that models tend to become complex. In the current study, log softmax with the ASGO is proposed, which approximates softmax. The proposed approach is based on the output of the model, making it acclimatized to the structure, parameters, and input of the model. The overall working of log softmax with the ASGO is given in Algorithm 2.
**Algorithm 2:** Log Softmax with ASGO.// Log Softmax Function 
Input:A vector of real numbers, denoted as x.

Output:A vector of the same shape as x, where each element represents the logarithm of the

softmax probability.

Notation:Let f(x) represent the log softmax function applied to vector x.

f(x)=log(softmax(x))

where softmax(x) is defined as:

$softmax(x_{i})=exp(x_{i})/sum(exp(x_{j}))\;for\;all\;j\;in\{1,2,...,n\}$

where n is the number of elements in the vector x.

Input→A vector of real numbers

 I→Iteration, K→logsoftmax

 $for\left(e:1\to I\right)do$

 for k:1→K do

 $for\;each\;input\;x_{j}^{n}\;in\left\{c_{j}\right\}do$

 $\;{CNN\;layer\;x}_{j}^{n}through\;CNN\;to\;obtain\;f_{j}^{n}$

 $\;{\max\;pool\;r}_{j,i}^{n}=\left(i-p_{i}\right)f_{j}^{n}$

 $Batch\;Normalizationd_{j,i}^{n}$

 $CNN\;layerr_{j,i}^{n}\;into\;AGSO$

 update all network parameters using log⁡ softmax

 if j=i then

$\;f_{j}^{n}$

 else

$\frac{1}{f_{j}^{n}}$ end

end

end

 calculate image classes

 if image classes classify then

 break

 end

 $\;\;f_{j}^{n}\;=\;log(softmax(x))$

   where softmax(x) is defined as:
 
$\;\;softmax(x_{i})\;=\;exp(x_{i})/sum(exp(x_{j}))\;for\;all\;j\;in\{1,2,...,n\}$

//Adaptive Subgradient Optimizer (Adagrad):

θ:Model parameters(weights and biases).

J(θ):The objective function to be minimized(typically the loss function).

$g_{t}:The\;gradient\;of\;the\;objective\;function\;with\;respect\;to\;\theta \;at\;time\;step\;t.$

η:Learning rate(a hyperparameter).

ε:A small constant to avoid division by zero.

$G:A\;diagonal\;matrix\;where\;each\;element\;G_{ii}accumulates\;the\;squared\;sum\;of\;past\;gradients$

$for\;parameter\;\theta_{i}.$

(3)$$G_{t}=G_{\left\{t-1\right\}}+g_{t}*g_{t}(element-wise\;squared\;sum\;accumulation)$$
(4)$$\theta_{t}=\theta_{\left\{t-1\right\}}-(\eta /sqrt(G_{t}+\varepsilon ))*g_{t}(element-wise\;division)$$

Note: The square root is applied element-wise to the matrix G_t_

Equations (3) and (4) are utilized for updating the model’s parameters iteratively. The Adagrad optimizer acclimatizes the learning rate for the individual parameters in accordance with the history of the gradients, providing minimal updating to the frequent parameters and maximal updating to infrequent parameters. This assists in the effectual optimization of the performance of the model. The note specifies the way Adagrad optimizer updates the parameters θ at time step t. 

## 4. Results and Discussion

The outcomes that were attained after execution of the proposed processes are discussed in this section, inclusive of dataset description, metrics considered for assessing the performance, empirical outcomes, performance, and comparative analysis.

### 4.1. Dataset Description

This study considered the DFU dataset accessible on Kaggle, which encompasses patient information about DFU. This dataset encompasses information like medical history, patient demographics, and details regarding foot ulcers. Data were gathered from individuals who were admitted to clinics in Amman from 2017 to 2019. This dataset is available from https://www.kaggle.com/datasets/laithjj/diabetic-foot-ulcer-dfu, accessed on 15 May 2023.

The dataset comprises 16 columns with 6803 entries. These columns encompassed patient ID, gender, age, hypertension, BMI, history of foot ulcer, smoking, fasting blood sugar, dyslipidemia, peripheral arterial disease, amputation history, diabetes duration, etc. The considered dataset could be valuable for medical experts researching DFUs and their related risk factors. It might also be utilized for developing prediction frameworks for identifying patients at maximum threat of developing DFUs.

Datasets including information such as history, patient demographics, and details regarding foot ulcers were considered. Patient demographics such as ID, gender, age, hypertension, BMI, history of foot ulcer, smoking, fasting blood sugar, dyslipidemia, peripheral arterial disease, etc., are considered to be some of the vital attributes in disease detection and diagnosis. This dataset can be valuable for medical experts researching DFUs and their related risk factors.

The dataset for inlaying the diabetic foot ulcer was collected from patients experiencing diabetic foot ulcers (DFUs) who were attending a multidisciplinary diabetic foot wound clinic on a regular basis. The medical history of the patients encompassing data such as HbA1c level and vascular status were examined when diagnosing DFU. Additionally, wound characteristics such as size, depth, location, infection status were also analyzed. Data were gathered from individuals admitted to an Amman clinic from 2017 to 2019.

### 4.2. Performance Metrics

The metrics that were considered when assessing the performance of the proposed system are discussed in this section.

#### 4.2.1. Accuracy

Accuracy is the computation of the overall correct classification rate and is represented by Equation (5):

(5) $$Accuracy_{A}=\frac{True_{Neg}+True_{Pos}}{True_{Neg}+False_{Neg}+False_{Pos}+True_{Pos}}$$

#### 4.2.2. Recall

Recall is defined as the proportion of relevant and retrieved images to the ratio of relevant images and is given by Equation (6):

(6) $$Recall_{R}=\frac{Relevant_{image}\cap Retrieved_{image}}{Relevant_{image}}$$

#### 4.2.3. Precision

Precision involves the computation of precise classification counts and is managed by incorrect classification rates. It is represented by Equation (7):

(7) $${Precision}_{P}=\frac{True_{Pos}}{True_{Pos}+False_{Pos}}$$

#### 4.2.4. F1-Score

The F1-score, also defined as the F-measure, is typically the harmonic mean of Recall (R) and Precision (P). It is mathematically given by Equation (8):

(8) $$F1\text{-}score=2*\frac{P*R}{P+R}$$

#### 4.2.5. AUC

AUC is stated as the precise integrated value that distinguishes the variations in classification and is given by Equation (9):

(9) $$AUC=\frac{1}{2}\left(\frac{True_{Pos}}{True_{Pos}+False_{Neg}}\right)+(\frac{True_{Neg}}{True_{Neg}+False_{Pos}})$$

### 4.3. EDA (Exploratory Data Analysis)

**Figure 4:**
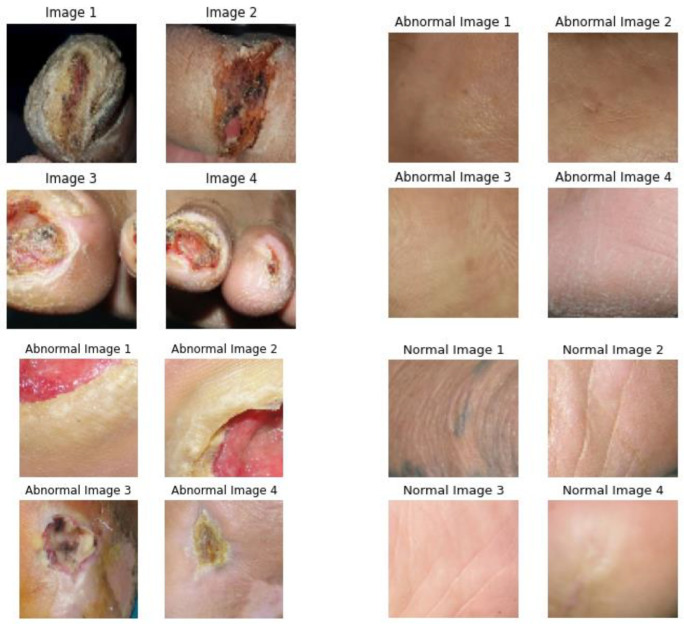
EDA for the considered dataset.

EDA indicates the crucial processes involved in undertaking initial analysis of the data for discovering patterns, spotting anomalies, hypothesis testing, and checking the assumptions with the assistance of graphical representations and summary statistics. The EDA corresponding to the considered dataset in the present study is shown in Figure 4.

### 4.4. Experimental Results

**Figure 5:**
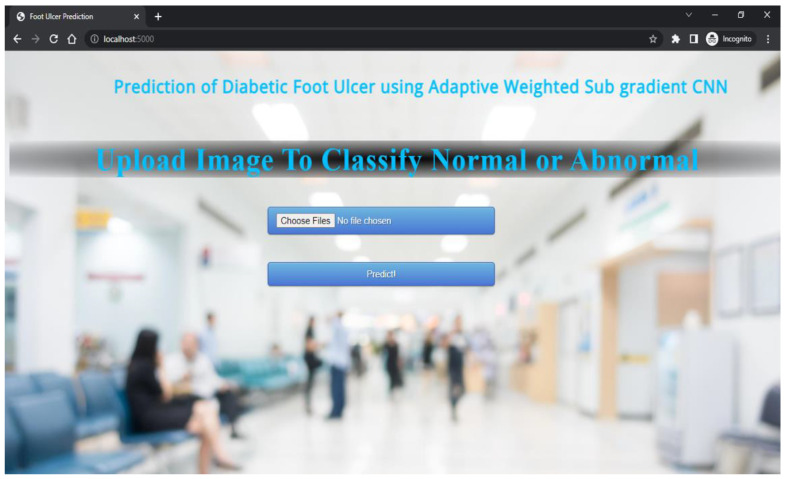
Upload of images for Classification.

The results that are displayed on the webpage are shown in this section. The upload of images for classification into abnormal or normal is shown in Figure 5.

**Figure 6:**
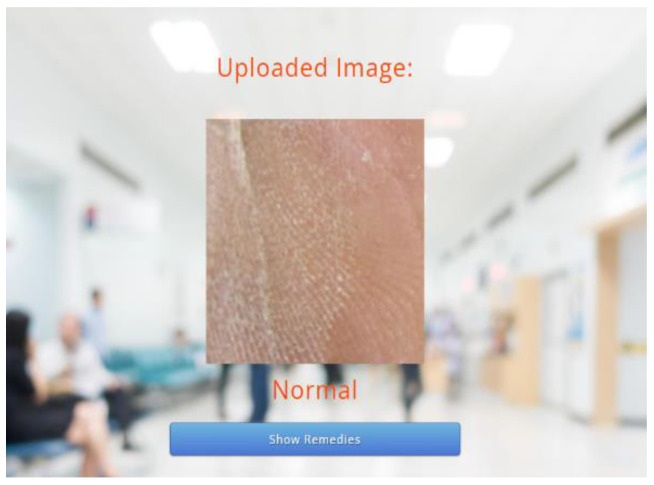
Predicted Results—Normal.

**Figure 7:**
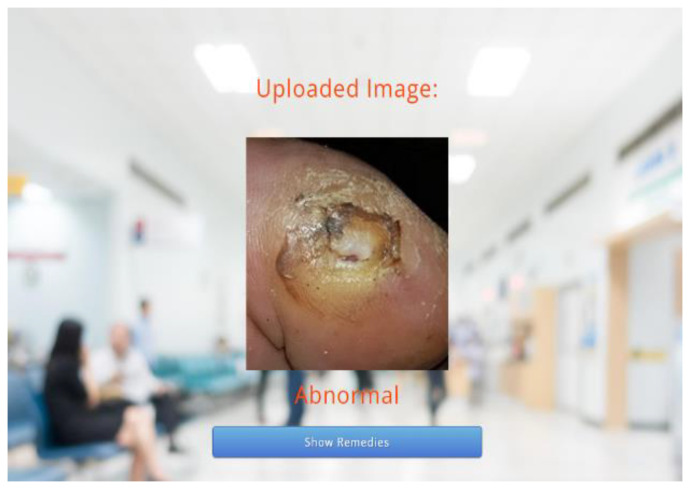
Predicted Results—Abnormal.

**Figure 8:**
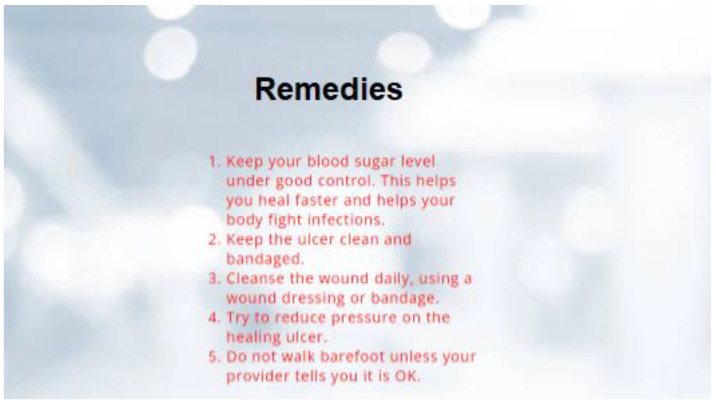
Remedies for Abnormal Cases.

Following this, the predicted results attained through the trained model are displayed on the webpage, as depicted in Figure 6 and Figure 7. As the predicted results in Figure 7 are abnormal, their equivalent remedies are shown in Figure 8.

**Figure 9:**
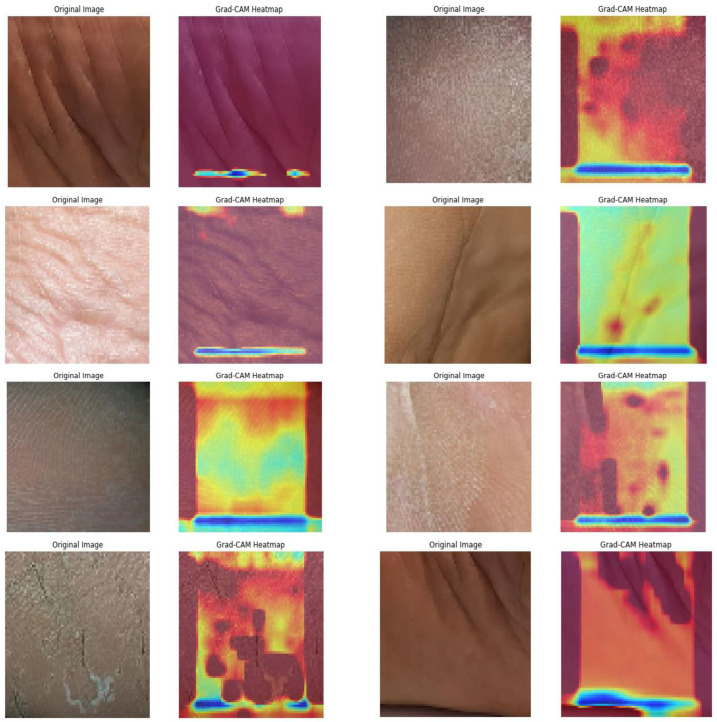

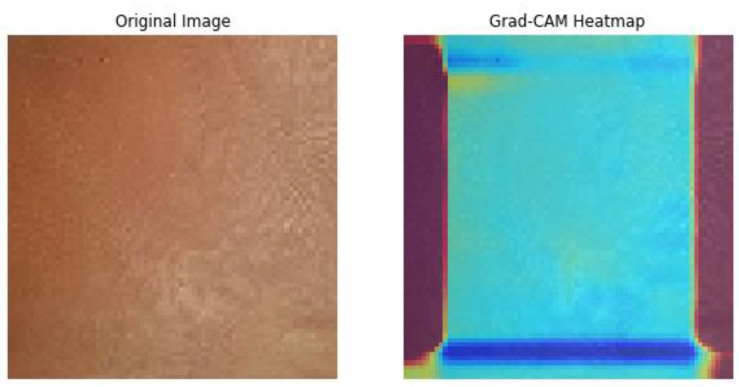
Normal GRADCAM Results.

**Figure 10:**
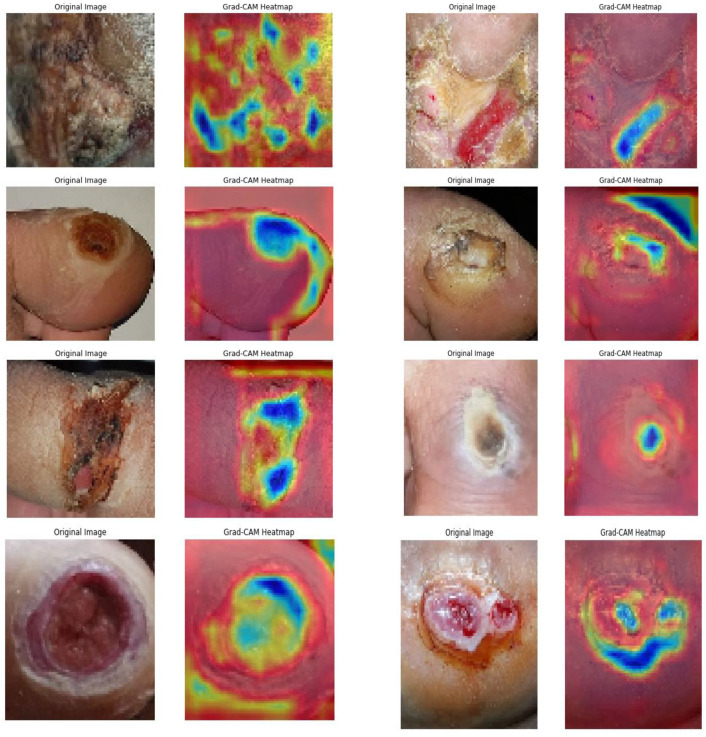

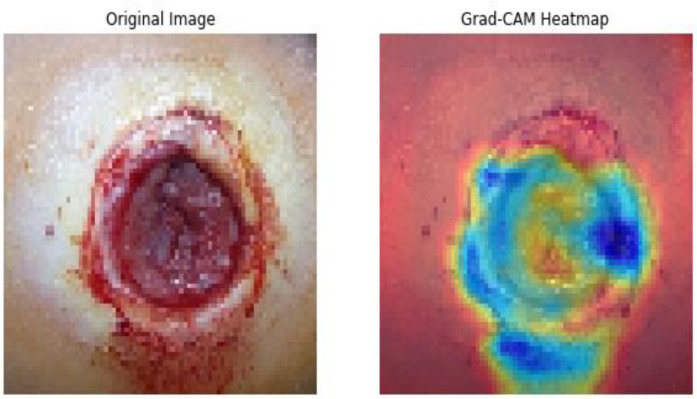
Abnormal GRADCAM Results.

Subsequently, the GRADCAM outcomes for normal cases are shown in Figure 9, whereas the GRADCAM results for abnormal cases are depicted in Figure 10. The significance and relevance of GRADCAM (Gradient-weighted Class Activation Mapping) are in visualizing the parts of an image that are most relevant to a particular class prediction made by a convolutional neural network (CNN). GRADCAM works by first computing the gradient of the CNN’s output score for the target class with respect to the features extracted from the image by the CNN’s convolutional layers. These gradients are then used to weight the feature maps from the CNN, and the resulting weighted feature maps are then summed to produce a heatmap that highlights the regions of the image that are most important for the CNN’s prediction. Typically, the GRADCAM method uses gradients of classification score in accordance with the overall convolution feature map for identification of the areas of the input, which highly influences the classification rate. Areas where this rate seems to be high are the suitable areas where the overall score relies most upon the data.

### 4.5. Performance Analysis

**Figure 11:**
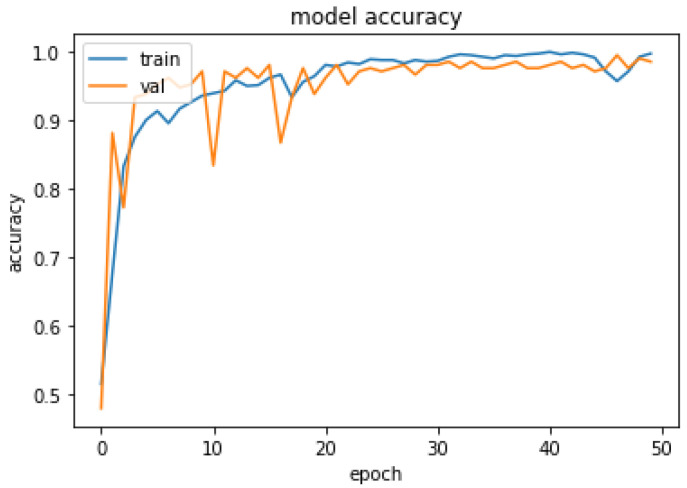
Evaluation of Accuracy.

**Figure 12:**
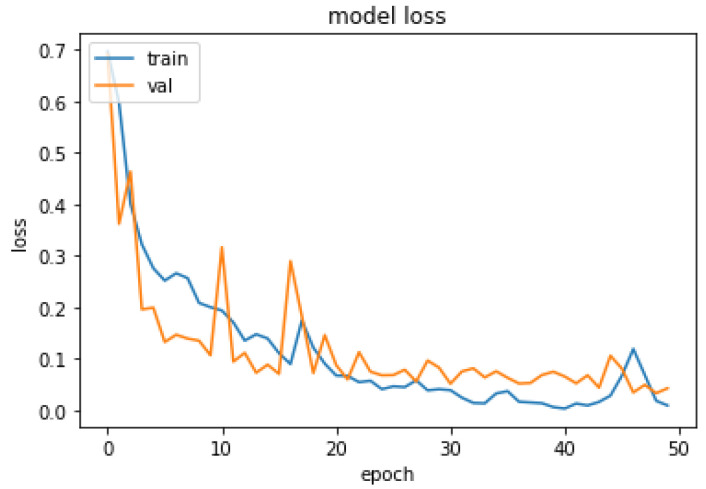
Evaluation about Loss.

The performance of the proposed system was evaluated in accordance with loss and accuracy; the evaluation in accordance with accuracy is shown in Figure 11, and the evaluation in accordance with loss is shown in Figure 12.

**Figure 13:**
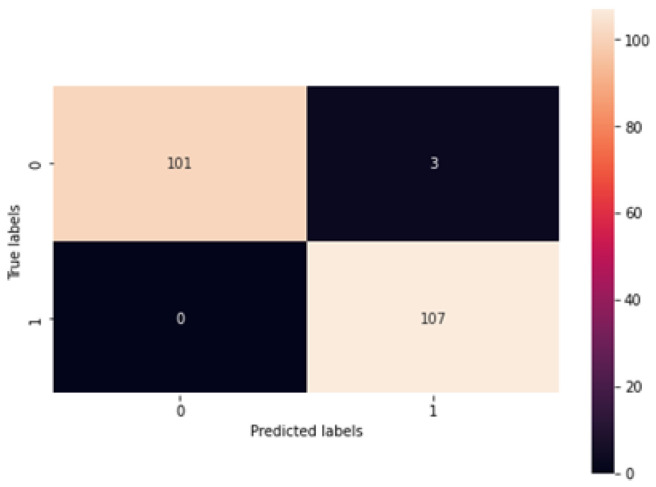
Confusion Matrix.

In Figure 11, training and validation accuracy seem to be similar at a high rate. Moreover, in Figure 12, it is found that the loss rate seems to be less, and the training and validation loss seems to correlate to the maximum extent. The low training loss indicates that the model is performing well on the training set, while a low testing loss indicates that the model is also performing well on new data. A high training loss indicates that the model is not learning well, while a high testing loss indicates that the model is not generalizing well to new data. Basically, the training loss will be lower than the testing loss, which is applicable in the proposed method, denoting the better performance of the model. The high accuracy and low loss revealed through the proposed system indicate its ideal performance. Following this, a confusion matrix was created to find the correct and misclassification rates. The corresponding results are shown in Figure 13.

**Figure 14:**
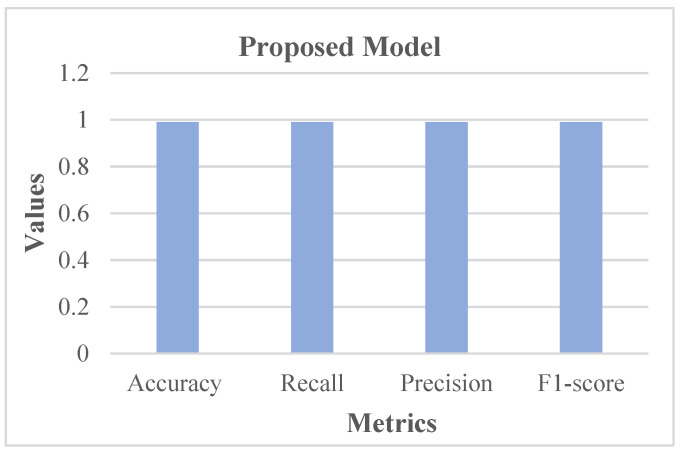
Analysis in accordance with Metrics.

Figure 13 shows that 101 normal classes were precisely classified as normal, 107 abnormal classes were correctly classified as abnormal, while 3 normal classes were misinterpreted as abnormal, and there was no misinterpretation of abnormal as normal. In this case, as the misclassification rate seems less than the correct classification rate, the proposed system is better. Subsequently, the proposed system was assessed using metrics like accuracy, recall, precision, and F1-score. The respective outcomes are shown in Table 1 with its equivalent graph in Figure 14.

The analytical outcomes, as depicted in Table 1 and Figure 14, revealed that the accuracy value was 0.99, the recall rate was 0.99, the precision rate was 0.99, and the F1-score rate was 0.99.

### 4.6. Comparative Analysis

**Figure 15:**
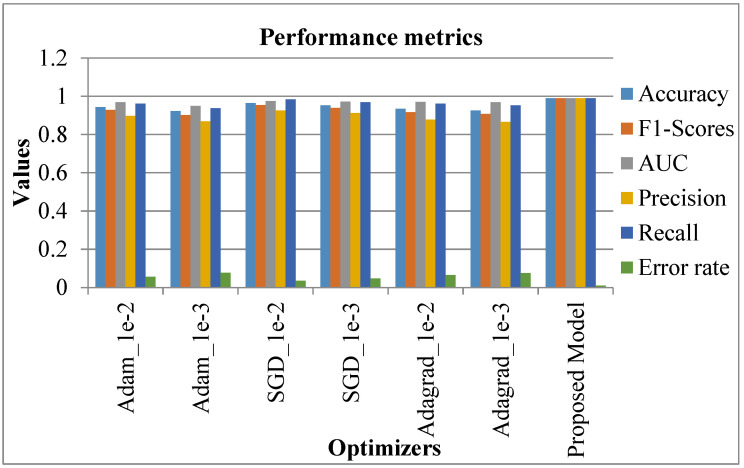
Analysis in accordance with Metrics [5].

The proposed system was comparatively assessed against conventional systems to confirm the better performance of the proposed system. The relevant results are discussed in this section. Initially, the comparison was accomplished regarding the metrics (accuracy, F1-score, AUC, precision, recall, and error rate). In this case, various optimization learning rates were considered, namely, Adam_1e-2, Adam_1e-3, SGD_1e-2, SGD_1e-3, Adagrad_1e-2, Adagrad_1e-3. The respective outcomes are shown in Table 2 with the equivalent graphical indication in Figure 15.

In Table 2 and Figure 15, existing systems like SGD_le_2 showed a high accuracy rate of 0.964, SGD_le_3 was 0.952, while Adam_le_2 achieved 0.944 as an accuracy rate. However, the proposed system showed a high accuracy rate of 0.99. Similarly, the F1-score, AUC, precision, and recall rate of the existing systems seemed to be better. Nevertheless, the proposed system showed a high performance, with 0.99 as the attained value.

**Figure 16:**
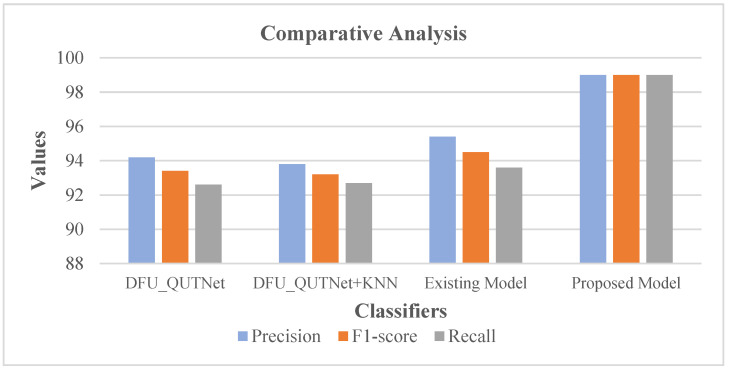
Analysis of Metrics [19].

On the contrary, the error rate of existing systems like SGD_le_2 was 0.036, SGD_le_3 had 0.048 as the error rate, while the proposed model showed a lower error rate of 0.01. In this case, as the proposed system showed maximum accuracy and a minimum error rate, the proposed system is better than the existing systems [5]. Following this, the precision, F1-score, and recall rates of the proposed system were comparatively validated with conventional methods encompassing the existing model [19], DFU_QUTNet, and DFU_QUTNet + KNN. The respective results are shown in Table 3 with the equivalent graph in Figure 16.

**Figure 17:**
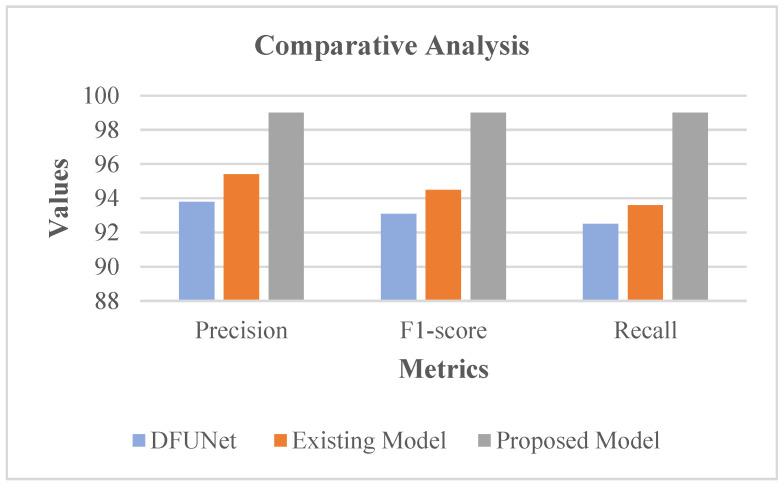
Evaluation in accordance with considered Metrics [19].

Table 3 and Figure 16 show that DFU_QUTNet achieved a 94.2% precision rate, DFU_QUTNet + KNN had 93.8% precision, while the existing model had 95.4% precision. Similarly, the recall and F1-score rates of the existing models were satisfactory. However, as an enhancement, the proposed system showed high precision, an F1-score, and a recall rate of 99%. Subsequently, the precision, F1-score, and recall rates of the proposed model were comparatively evaluated against traditional models encompassing the existing model [19] and DFUNet. Respective outcomes are presented in Table 4 with the equivalent graph in Figure 17.

Table 4 and Figure 17 show that DFUNet had a 93.8% precision rate, while the existing model had 95.4% precision. Similarly, the recall and F1-score rates of the conventional models were acceptable. Nonetheless, as an improvement, the proposed system revealed high precision, F1-score, and a recall rate of 99%. In the proposed classifier phase, RIW possessed the innate ability for effectual learning of the shift of feature space among the convolutional layers. Moreover, for evading the gradient underflow, the log softmax function was used.

Additionally, with the usage of log softmax ASGO for the activation function, the gradient steps are controlled. Moreover, the adaptive modification of the proximal function simplifies the learning rate. Due to all these merits, the proposed system is capable of performing ideal classification, and hence, it is capable of producing optimal outcomes, which was confirmed through the analysis of recent conventional models.

## 5. Conclusions

This study endeavored to classify DFU images into normal or abnormal using the proposed AWSg-CNN. Further, the study deployed a Flask model to easily create web applications to predict foot ulcers and show remedies in abnormal cases. The overall performance of the study was assessed by comparison with traditional methods to reveal the ideal performance of the model in DFU classification. In this case, performance was assessed by accuracy, loss, and a confusion matrix. The data from clinical trials were demonstrated in various aspects of clinical trials, and the effectiveness of this technique was projected. These data were used in the proposed technique to improve patient outcomes, such as reducing the risk of complications and improving patient satisfaction. And it could be more accurate than other methods, which can reduce the risk of misdiagnosis.

A high accuracy rate and low loss rate were attained. Moreover, from the confusion matrix, it was found that correct classification rates outnumbered misclassification rates, confirming the efficacy of the proposed system. Further, a comparative analysis was undertaken to prove the effectual performance of the present method over conventional methods. To confirm this, various metrics were considered, namely, accuracy, recall, precision, F1-score, AUC, and error rate. Some of the limitations of the proposed system were described and highlighted for reference. During random initialization the weights were initialized to random, which were either large or too small; thus, the network may not be able to learn effectively. The number of hyperparameters that need to be tuned will have a significant impact on the overall performance of the network. An Adaptive Sub-gradient Optimizer that gets stuck in local optima or saddle points will fail in exploring the global optimum. Overall analytical results confirmed that the proposed system showed a high accuracy rate of 99% with a minimum error rate of 0.01. The high accuracy value attained by the proposed system makes it highly probable to execute in real time. Further, the algorithm can be utilized in other DFU datasets. As a part of future work, it will be necessary to clearly understand the pathogenesis of DFUs as it is the cornerstone of curing this condition. Only by accurately comprehending the regulation of the pathogenesis of DFUs can new drugs or interventions for relevant targets be developed. The use of stem cells in treating DFUs is emerging as a promising treatment. Embryonic and mesenchymal stem cells can be induced to differentiate into myofibroblasts, keratinocytes, and endothelial cells, which are components of wound healing.

## Figures and Tables

**Table 1:** Analysis of Metrics.

	Accuracy	Recall	Precision	F1-Score
Proposed Model	0.99	0.99	0.99	0.99

**Table 2:** Analysis of Metrics [5].

Optimization Learning Rate	Accuracy	F1-Score	AUC	Precision	Recall	Error Rate
Adam_1e-2	0.944	0.928	0.968	0.897	0.961	0.056
Adam_1e-3	0.922	0.902	0.95	0.869	0.937	0.077
SGD_1e-2	0.964	0.954	0.974	0.926	0.984	0.036
SGD_1e-3	0.952	0.939	0.972	0.912	0.968	0.048
Adagrad_1e-2	0.934	0.917	0.97	0.878	0.961	0.066
Adagrad_1e-3	0.925	0.907	0.968	0.865	0.953	0.075
Proposed Model	0.99	0.99	0.99	0.99	0.99	0.01

**Table 3:** Analysis of Metrics [19].

Classifier	Precision	F1-Score	Recall
DFU_QUTNet	94.2	93.4	92.6
DFU_QUTNet + KNN	93.8	93.2	92.7
Existing Model	95.4	94.5	93.6
Proposed Model	99	99	99

**Table 4:** Analysis with regard to Metrics [19].

Network	Precision	F1-Score	Recall
DFUNet	93.8	93.1	92.5
Existing Model	95.4	94.5	93.6
Proposed Model	99	99	99

## Data Availability

Not applicable.
